# Gemcitabine-Based Neoadjuvant Treatment in Borderline Resectable Pancreatic Ductal Adenocarcinoma: A Meta-Analysis of Individual Patient Data

**DOI:** 10.3389/fonc.2020.01112

**Published:** 2020-08-11

**Authors:** Francesco Giovinazzo, Fiammetta Soggiu, Jin-Young Jang, Eva Versteijne, Geertjan van Tienhoven, Casper H. van Eijck, Youngmin Han, Seong Ho Choi, Chang Moo Kang, Mark Zalupski, Hasham Ahmad, Sarah Yentz, Scott Helton, J. Bart Rose, Chie Takishita, Yuichi Nagakawa, Mohammad Abu Hilal

**Affiliations:** ^1^Department of Surgery, University Hospital of Southampton NHS Foundation Trust, Southampton, United Kingdom; ^2^Hepato-Pancreato-Biliary and Liver Transplant Unit, Royal Free Hospital, London, United Kingdom; ^3^Department of Surgery, Seoul National University Hospital, Seoul, South Korea; ^4^Department of Radiation Oncology, Cancer Center Amsterdam, Amsterdam UMC, University of Amsterdam, Amsterdam, Netherlands; ^5^Department of Surgery, Erasmus MC Cancer Institute, Rotterdam, Netherlands; ^6^Department of Surgery, Sungkyunkwan University School of Medicine, Seoul, South Korea; ^7^Division of HBP Surgery, Department of Surgery, Yonsei University College of Medicine, Seoul, South Korea; ^8^Department of Medicine, University of Michigan, Ann Arbor, MI, United States; ^9^Department of Surgery, University Hospital of Leicester NHS Trust, Leicester, United Kingdom; ^10^Section of General, Thoracic and Vascular Surgery, Department of Surgery, Virginia Mason Medical Center, Seattle, WA, United States; ^11^Section of Surgical Oncology, University of Alabama, Birmingham, AL, United States; ^12^Department of Gastrointestinal and Pediatric Surgery, Tokyo Medical University, Tokyo, Japan; ^13^Depatment of Surgery, Fondazione Poliambulanza Istituto Ospedaliero Multispecialistico, Brescia, Italy

**Keywords:** gemcitabine, gemcitabine-based neoadjuvant, neoadjuvant treatment of pancreatic cancer, Pancreatic ductal adenocancinoma, Borderline resectable pancreaic adenocarcinoma

## Abstract

**Background:** Non-randomized studies have investigated multi-agent gemcitabine-based neo-adjuvant therapies (GEM-NAT) in borderline resectable pancreatic ductal adenocarcinoma (BR-PDAC). Treatment sequencing and specific elements of neoadjuvant treatment are still under investigation. The present meta-analysis aims to assess the effectiveness of GEM-NAT on overall survival (OS) in BR-PDAC.

**Patients and Methods:** A meta-analysis of individual participant data (IPD) on GEM-NAT for BR-PDAC were performed. The primary outcome was OS after treatment with GEM-based chemotherapy. In the Individual Patient Data analysis data were reappraised and confirmed as BR-PDAC on provided radiological data.

**Results:** Six studies investigating GEM-NAT were included in the IPD metanalysis. The IPD metanalysis was conducted on 271 patients who received GEM-NAT. Pooled median patient-level OS was 22.2 months (95%CI 19.1–25.2). R0 rates ranged between 81 and 95% (*I*^2^ = 0%, *p* = 0.64), respectively. Median OS was 27.8 months (95%CI 23.9–31.6) in the patients who received NAT-GEM followed by resection compared to 15.4 months (95%CI 12.3–18.4) for NAT-GEM without resection and 13.0 months (95%CI 7.4–18.5) in the group of patients who received upfront surgery (*p* < 0.0001). R0 rates ranged between 81 and 95% (*I*^2^ = 0%, *p* = 0.64), respectively. Overall survival in the R0 group was 29.3 months (95% CI 24.3–34.2) vs. 16.2 months (95% CI 7·9–24.5) in the R1 group (*p* = 0·001).

**Conclusions:** The present study is the first meta-analysis combining IPD from a number of international centers with BR-PDAC in a cohort that underwent multi-agent gemcitabine neoadjuvant therapy (GEM-NAT) before surgery. GEM-NAT followed by surgical resection improve survival and R0 resection in BR-PDAC. Also, GEM-NAT may result in a good palliative option in non-resected patients because of progressive disease after neoadjuvant treatment. Results from randomized controlled trials (RCTs) are awaited to validate these findings.

## Introduction

Pancreatic ductal adenocarcinoma (PDAC) is a not uncommon lethal malignancy, with a 5-year survival of 8% for all the stages. Patients undergoing resection have 20% survival rate at 5-years which may be as high as 32% in case of complete resection and 40% in the subgroup with node-negative disease. Only a minority of patients, however, are eligible for surgery at the time of diagnosis, due to metastatic or locally advanced disease (1, 2).

A borderline resectable (BR)-PDAC is a tumor with a variable degree of vascular contact or involvement that may not permit a complete resection without vascular resection and/or reconstruction, making resection challenging, although technically possible. Various definitions of BR-PDAC have been proposed but the literature supports that this group of patients does benefit from surgical resection if a complete resection (R0) can be reached (3, 4). In addition, recent studies have suggested that the use of a multimodal neoadjuvant treatment approach may better select the patient that will benefit from surgery by increasing the R0 resection rate and improving overall survival (5–7). Previous studies have focused on the benefit of neoadjuvant treatment compared to upfront surgery in PDAC, but there is paucity of evidence on long term outcomes in BR-PDAC (7).

To date no standard neoadjuvant protocol has been agreed in BR-PDAC (8–10). Palliative gemcitabine has been a standard of care in locally advanced or metastatic PDAC for many years and is used alone or in multi-agent combinations, and in association with radiotherapy (6). The type of treatment choice is influenced by individual patient features and status and based on data available from metastatic PDAC.

The present meta-analysis of individual participant data (IPD) aimed to assess the impact of the use of neoadjuvant therapies with Gemcitabine based protocols in patients with borderline resectable PDAC. The primary outcome of the meta-analysis was overall survival (OS) in patients who received GEM-NAT followed by resection.

## Methods

### Search Strategy

The systematic review and meta-analysis were conducted in accordance to the Preferred Reporting Items for Systematic Reviews and Meta-Analyses (PRISMA) guidelines.

A computerized search of PubMed, Embase, Ovid Medline, and Cochrane Library was carried out. Articles published from time of inception to February 2020 were included. An advanced search was performed with the following search mesh terms: “pancreatic neoplasm and neoadjuvant therapy.” Reference lists of all obtained and relevant articles were screened manually and cross-referenced to identify any additional studies. The site clinicaltrials.gov was interrogated for any ongoing or concluded trial on the topic with available results.

### Outcomes of Interest

The primary outcome was median overall survival (OS) in patients with BR-PDAC treated with Gemcitabine-based neo-adjuvant treatments (GEM-NAT) with or without surgical resection. Secondary outcomes were: complete resection (R0) and resection rate. In the IPD analysis all stages were re-assessed and confirmed as BR-PDAC according to NCCN guidelines based on provided radiological data.

### Inclusion Criteria

Studies that reported results on patients diagnosed with BR-PDAC with the outcome of interest were included in the review. Studies including both patients with BR-PDAC and with locally advanced PDAC were only included when the primary outcome was available for the separate cohort of BR-PDAC. Only data from the centers who provided anonymized individual patient data with results on GEM-NAT were included in the IPD meta-analysis. Articles focused on pancreatic neuroendocrine neoplasia or other histology were excluded. When two or more articles were reported from the same institution and/or author, the one of higher quality or the most recent publication was included in the analysis.

Abstracts, letters, comments, editorials and expert opinions, unpublished articles and abstracts, reviews without original data, case reports were excluded from the meta-analysis.

Two reviewers (FS and HA) independently screened the titles and abstracts of all retrieved articles. The full texts of articles with the potential to fulfill the inclusion criteria were obtained and checked for eligibility. The following information was extracted from each article: first author, year of publication, study design, study population characteristics, number of subjects treated, type of neo-adjuvant treatment, dropout rate, procedure-related mortality and morbidity, median and disease-free survival complete oncological resection (R0), and resection rate.

After selecting eligible articles for meta-analysis, contact details of authors were gathered from recent articles or the internet and authors were asked to collaborate with us. A second request was sent to non-responders 4 weeks later. Authors who agreed to collaborate were requested to provide anonymous IPD for clinico-pathological and radiological characteristics, treatment, postoperative, and long term outcomes.

### Data Analysis

The meta-analysis was performed using R software suite (v3.4.0, https://www.R-project.org), and Kaplan Meier curves were calculated with SPSS (v24, Chicago, IL, USA). Pooled effect was calculated using either the fixed effects or the random effects model. Time-to-event methods were used for the median survival. Hazard Ratio (HR) was derived from ln(HR) and Standard Error (SE). Studies not reporting *p*-value for survival analysis were excluded (11). Statistical heterogeneity between trials was evaluated by χ^2^ and *I*^2^, with significance being set at *p* ≤ 0.10 (12). In the absence of statistically significant heterogeneity, the fixed-effect method was used to combine the results. When heterogeneity was confirmed (*p* ≤ 0.10), the random-effect method was used. Potential publication bias was investigated by funnel plot, Egger's test, was used to assess funnel plot asymmetry (13) and Makaskill's test was used to quantify the bias (14). *P* < 0.050 (two-tailed) was considered to indicate statistical significance. The methodological quality of the individual studies was assessed with the Critical Appraisal Skill Program (CASP) tool (15).

## Results

### Literature Search

**Figure 1:**
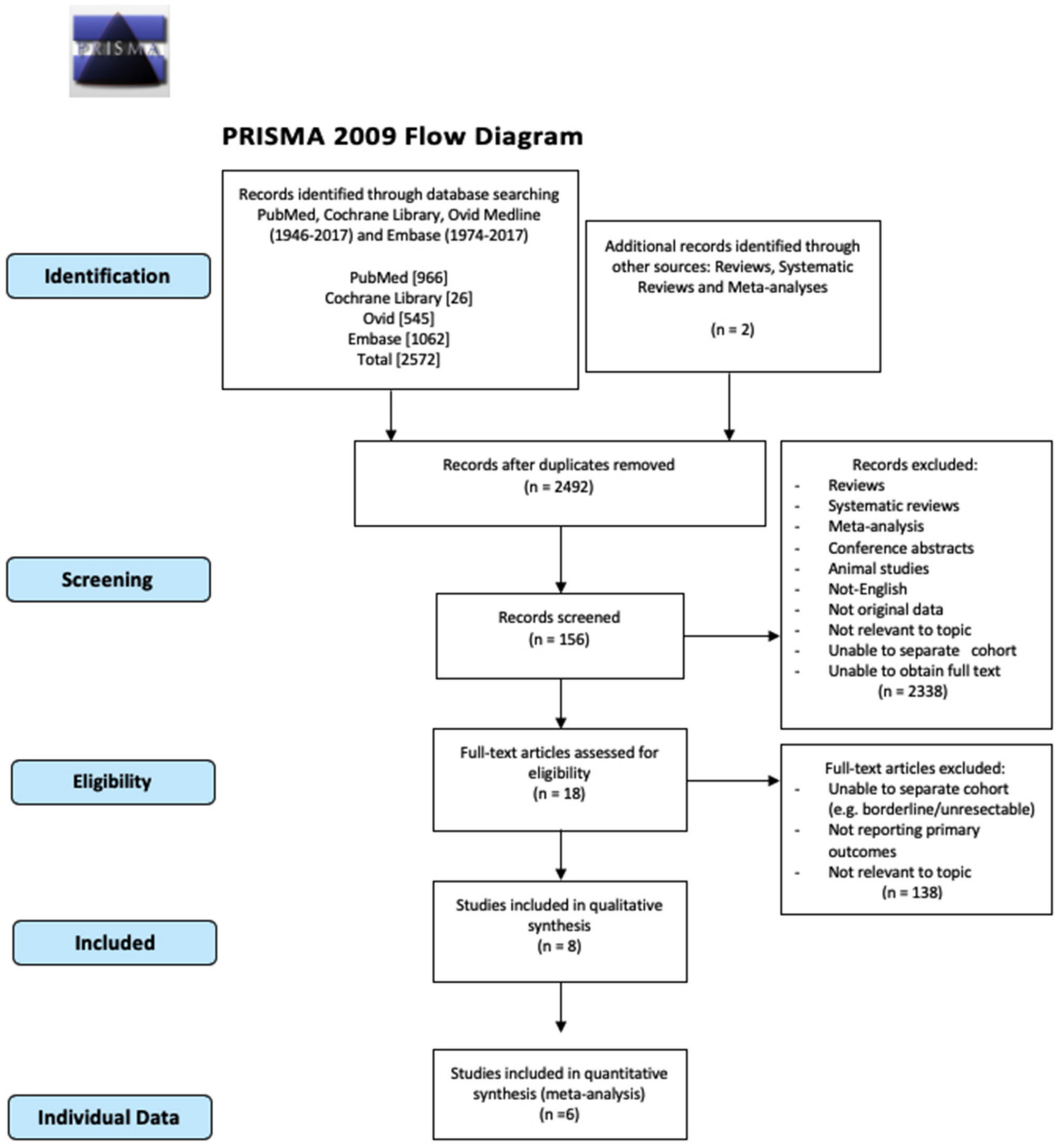
PRISMA 2009 flow diagram.

The number of studies screened, assessed, and excluded is reported in the PRISMA flow diagram (Figure 1). Eighteen full text articles were assessed for eligibility and six studies provided data at an IPD (Table 1) (7, 16–20).

**Table 1:** Studies included in the individual patient data (IPD) meta-analysis.

**Author**	**Year**	**Study design**	**Neoadjuvant treatment**	**Median F/U (months)**	**Treatment**	***N***	**Resection rate (%)**	**R0 resection (%)**	**Median OS (months)**	**Median DFS (months)**
Rose JB.	2014	Retrospective	Gem + Docetaxel +/– RT 50.4 Gy	21.6	NAT/resection	31	48.0	87.0	*NR*	23.2
					NAT/no resection	33			15.4	
Kim EJ.	2013	Prospective	Gem + Ox + RT 30 Gy	31.4	NAT/resection	28	93.3	-	25.4	-
					NAT/no resection	11			10.9	
Lee JH	2015	Prospective	Gem +/– Cisplatinum +/– Cap +/– 45 or 50.4 Gy	-	NAT/resection	30	100	93.3	30.9	-
					NAT/no resection	12			19.5	
Nagakawa Y.	2017	Prospective	Gem + S1+ RT 50.4Gy	20.0	NAT/resection	19	76.0	94.7	22.9	-
					NAT/no resection	6			9.3	
Jang JY	2018	RCT	Gem + RT 45 + 9 Gy	-	NAT/resection	17	51.8%	82.3	21.0	-
					NAT/no resection	10			-	
Versteijne E	2020	RCT	Gem + RT 36 Gy (total)	27.0	NAT/resection	25	61.0	71.0	16.0	8.1
					NAT/no resection	29			-	

*NAT, Neoadjuvant Treatment; Cap, Capecitabine; Gem, Gemcitabine; Ox, Oxaliplatin; 5-FU, 5-Fluorouracil; RT, Radiotherapy; FDR, Fixed-Dose Rate; NR, Not Reached; RCT, Randomized controlled trial*.

### Individual Patient Data Meta-Analysis

**Figure 2:**
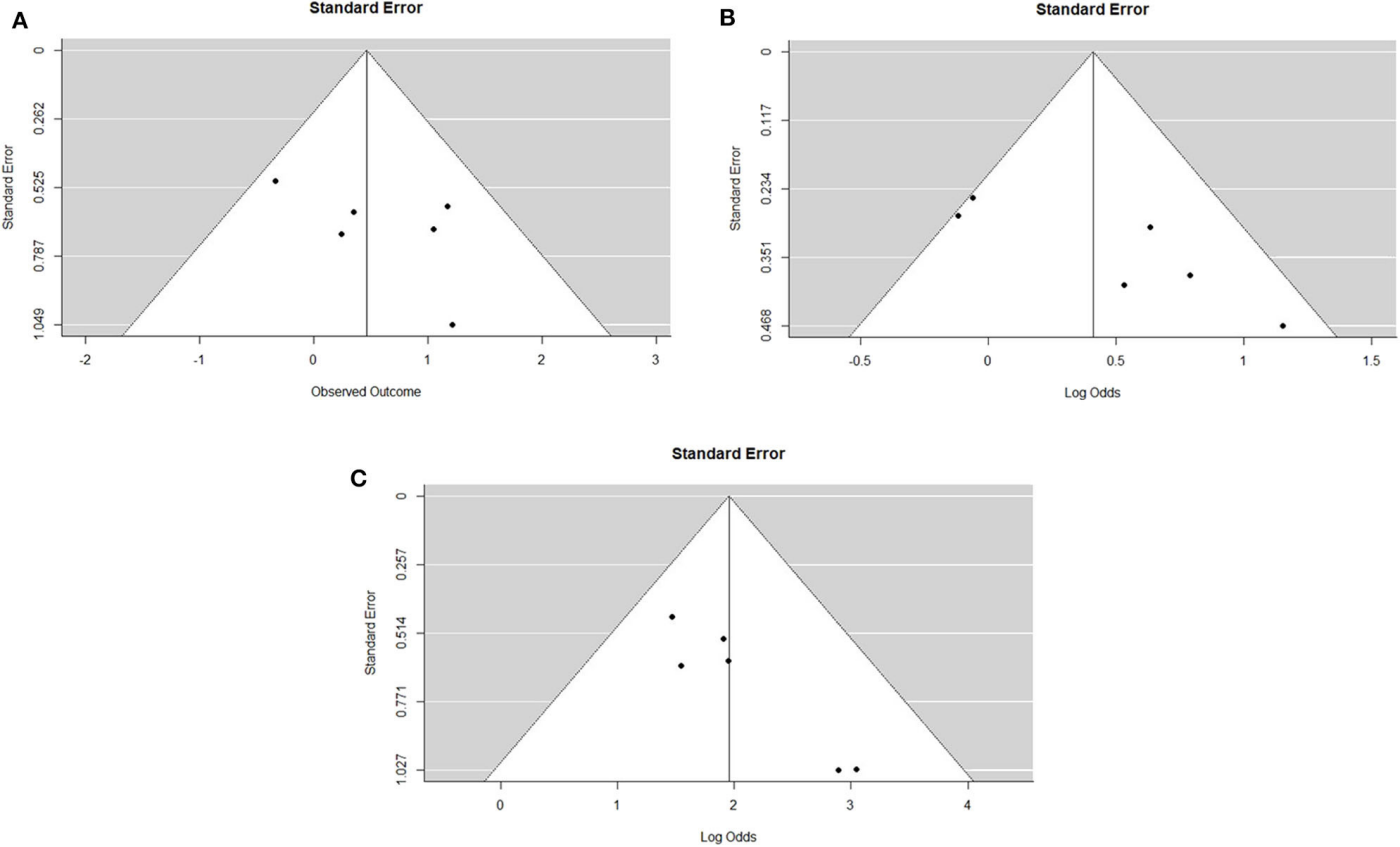
**(A)**. Funnel plot for survival outcomes. Test for funnel plot asymmetry: *t* = 1.4439, df = 4, *p* = 0.2223. **(B)**. Funnel plot for resection outcomes. Test for funnel plot asymmetry: *t* = 3.2400, df = 4, *p* = 0.0317. **(C)**. Funnel plot for RO outcomes. Test for funnel plot asymmetry: *t* = 4.3507, df = 4, *p* = 0.0122.

**Figure 3:**
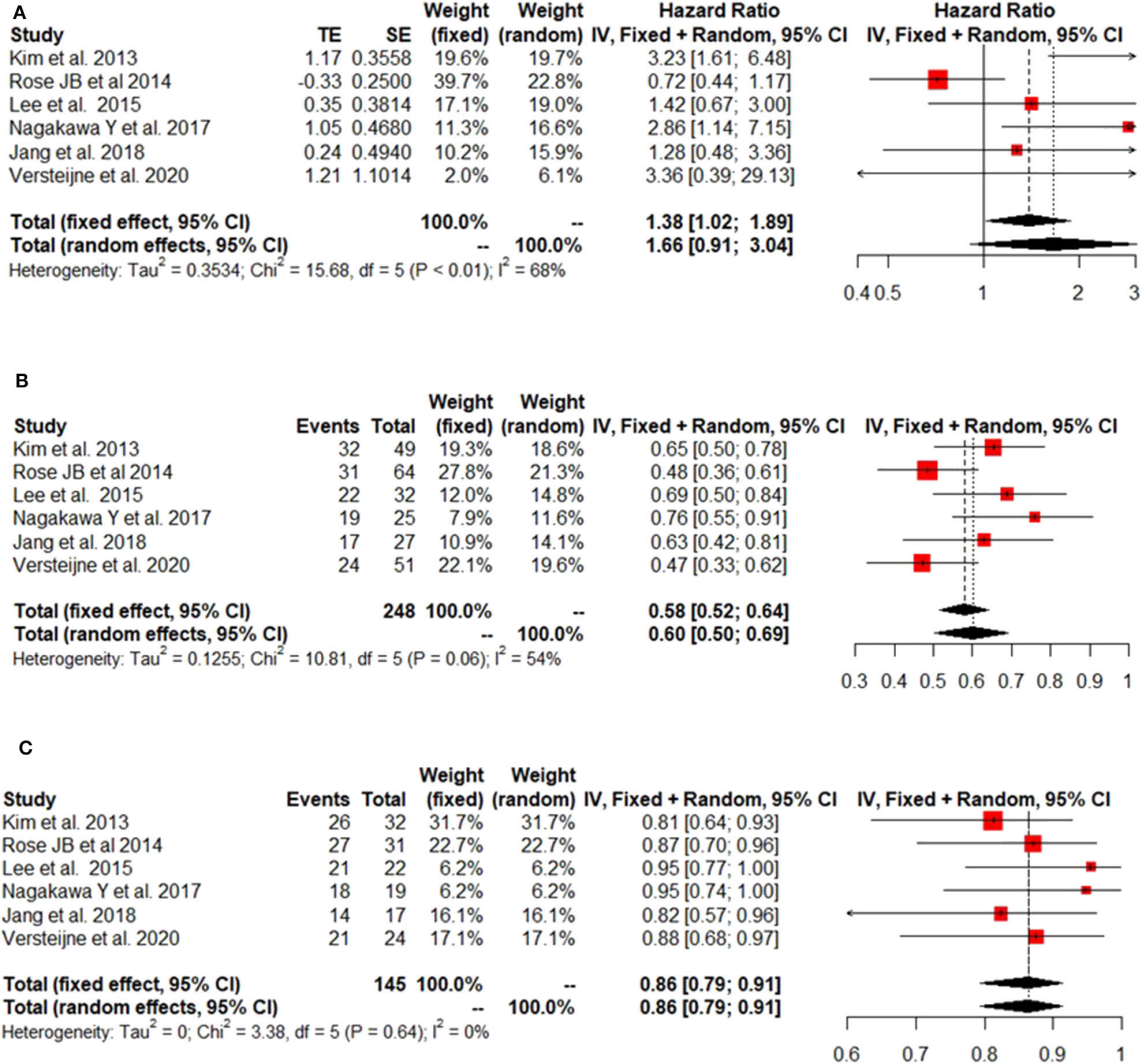
Forest plots showing HR of survival **(A)**, rates of Resection Rate **(B)** and RO **(C)**.

The IPD metanalysis was conducted on the data provided on 271 patients who received GEM-NAT. The included studies showed evidence of an asymmetrical distribution in overall survival (Test for funnel plot asymmetry: *t* = 1.4439, df = 4, *p* = 0.2223) (Figure 2A), resection rate (Test for funnel plot asymmetry: *t* = 3.2400, df = 4, *p* = 0.0317) (Figure 2B) and R0 rate (Test for funnel plot asymmetry: *t* = 4.3507, df = 4, *p* = 0.0122) (Figure 2C). There was no heterogeneity differences with regards to resection rate, which ranged from 47 to 76% (random effect model 0.60 (95% CI 0.50–0.69) *I*^2^ = 54%, *p* = 0.06) (Figure 3A), R0 rate, which ranged from 81 to 95% (random effect model 0.86, 95% CI 0.79–0.91, *I*^2^ = 0%, *p* = 0.64) (Figure 3B).

**Figure 4:**
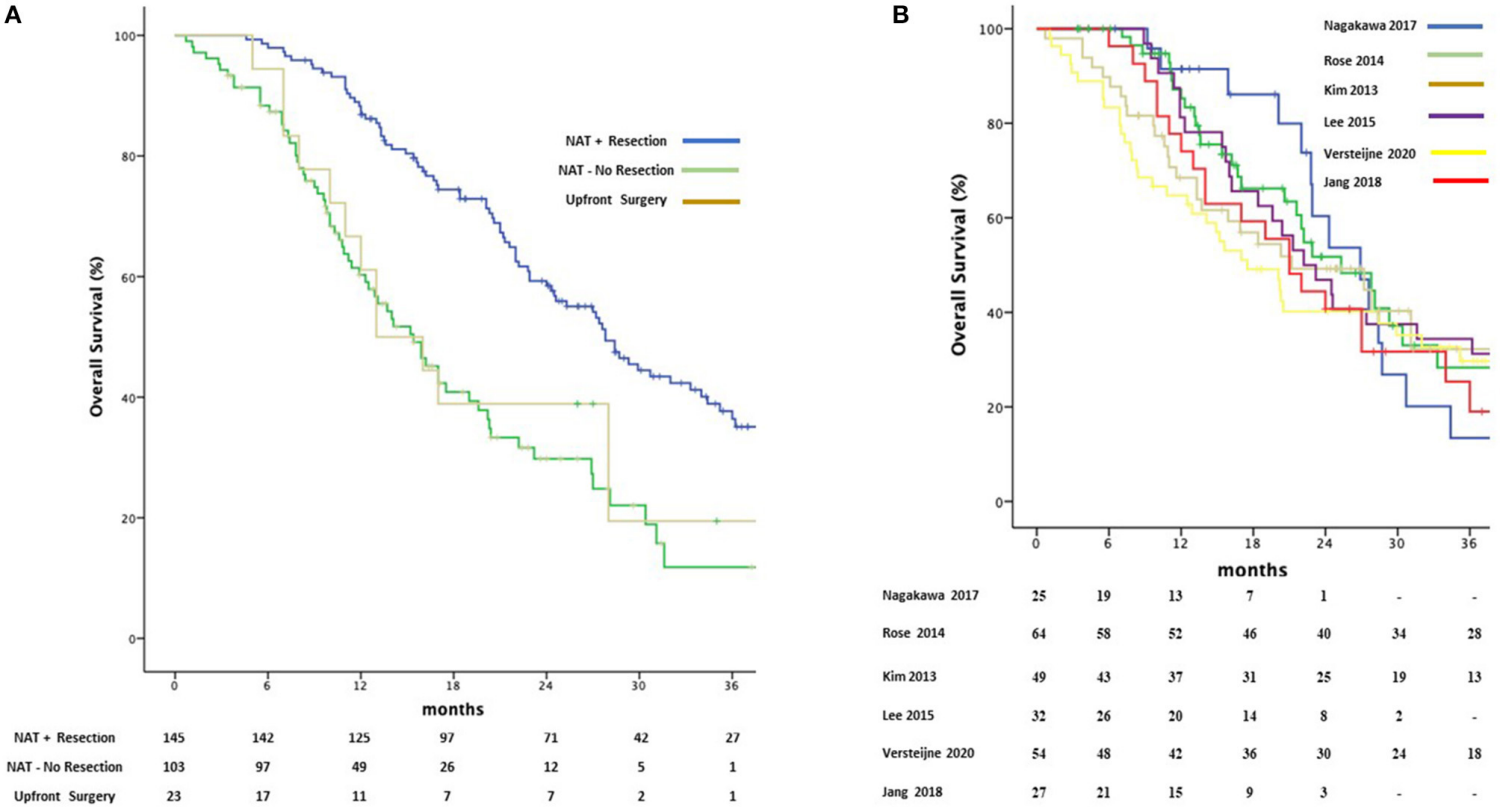
Kaplan-Meier curves for pooled overall survival in patients receiving NAT-GEM following by resection and patients who received GEM-NAT but did not undergo resection or underwent upfront surgery **(A)** and amongst Treatment Protocols **(B)**.

Depending on the different centers' protocols, Gemcitabine was used as single agent in 110 (40.6%) patients or in combination with Docetaxel (67 patients, 24.7%) oxaliplatin (53 patients, 19.5%) S-I (25 patients, 9.2%) or other agents such and Cisplatin, Capecitabine and 5FU. Median OS was 27.8 months (95%CI 23.9–31.6) in the patients who received NAT-GEM followed by resection compared to 15.4 months (95%CI 12.3–18.4) for NAT-GEM without resection and 13.0 months (95%CI 7.4–18.5) in the group of patients who received upfront surgery from the study published by Jang et al. (Figure 4A) (7). Patients receiving a multimodality treatment with NAT-GEM followed by surgery had a significantly better OS compared to both patients who did not complete the treatment with surgery and the patients who had upfront surgery (*p* = 0.000)?. Median OS for NAT-GEM +/– resection in the single studies included in the IPD analysis was similar (*p* = 0.813): 26.9 months (95%CI 21.1–32.6) for Nagakawa et al. (16), 25.3 months (95%CI 18.3–32.2) for Rose et al. (18), 21.2 months (95%CI 8.6–33.7) for Kim et al. (19), 22.2 (95%CI 16.5–27.8) for Lee et al. (20), 17.5 months (95%CI 10.9–24.0) for Versteijne et al. (17) and 22.9 months (18.8–26.7) for Jang et al. (7) (Figure 4B).

Data on radiological response following NAT-GEM was available for 200 patients, of which 76 (38%) achieved downstaging. Median OS was significantly improved in patients who achieved downstaging (28.7 months 95%CI 22.0–35.3 vs. 22.0 months; 95%CI 18.6–25.3; *p* = 0.026) (Supplementary Figure 1).

Median disease-free survival (DFS) in the entire cohort was 11.8 (95%CI 9.3–14.3). Data on toxicity was available for 244 patients: 27 (11.2% did not experience toxicity from GEM-NAT, 139 patients (56.9%) had toxicity grade I-II, whilst toxicity III and IV was seen in 92 (37.7%) and 20 (8.2%) of the patients.

Information on oncological complete resection was available for 158 patients who underwent resection following GEM-NAT. R0 resection was achieved in 130 patients (82.3%) with a median OS of 29.3 months (95% CI 24.3–34.2) vs. 16.2 months (95% CI 7.9–24.5) in the R1 group (*p* = 0.001).

## Discussion

Different strategies combining surgery and chemoradiotherapy are being investigated for BR- PDAC (21). Although neoadjuvant therapy had shown beneficial effects in BR-PDAC by improving overall survival and the rate of R0 resection, treatment sequencing and specific elements of neoadjuvant treatment are still under investigation (17, 22–24). The benefit of gemcitabine-based combination therapies is hotly debated and reported results have shown conflicting conclusions (25–27). A previous meta-analysis of 20 phase III RCT (*n* = 6,296) has shown no differences in overall survival between single agent gemcitabine and combination gemcitabine therapy in inoperable pancreatic cancer (RR 0.93, 95% CI 0.84–1.03, *p* = 0.17) (25). Therefore, the evidence to recommend a specific neoadjuvant regimen is limited and practices vary with regard to the use of combination chemotherapy and/or radiotherapy (8–10). This meta-analysis of different gemcitabine-based protocols has shown similar results to other reported neoadjuvant regimens (5), with a pooled median patient-level OS of 27.2 (95% CI 23.0–31.3) months in resected patients.

Up front surgery for BR-PDAC is still considered an option in some centers (28, 29). The number of RCTs comparing neoadjuvant therapy in BR-PDAC vs. up front surgery are very limited and the outcomes of different regimens still unknown (9, 30). Recently, OS in a RCT was significantly better in the gemcitabine-based neoadjuvant chemoradiation treatment than in the upfront surgery group (21 vs. 12 months *p* = 0.028) and the results has been confirmed by the present meta-analysis (7). The Dutch Pancreatic Cancer Group reported in a randomized multicenter phase III trial a better OS in the preoperative gemcitabine arm than in the immediate surgery group (median 13.5 vs. 17.1 months; HR 0.71; *p* = 0.074) (17). In the present meta-analysis, the group of patients that did not proceed to surgery had similar median OS [20.4 (95% CI 12.7–28.0)] to the upfront surgery group in these two RCTs. Therefore, we speculate that this result confirms the findings and supports the use of neoadjuvant therapy to select patients with a favorable biological disease and to provide a palliative option in the non-responder or unresected groups.

Studies have demonstrated that neoadjuvant treatment does not decrease the rate of surgical resection in BR-PDAC and may lower surgical complication rates (31–35). This is likely due to a selection bias for healthier patients that are able to complete neoadjuvant treatment. Chemotherapy combinations are likely more efficacious in the neoadjuvant setting but are associated with increased toxicities. In advanced disease, gemcitabine has less Grade 3–4 toxicity as compared to FOLFIRINOX. As NAT, in one phase II study, a significant number of patients (23%) did not proceed to surgery due to toxicities or poor performance status following neoadjuvant gemcitabine and oxaliplatin (34). The present results seem to confirm that Gem-based chemotherapy is better tolerated than multidrug regimens with a minimal drop-out rate not related to progression disease.

Neoadjuvant therapy has permitted tumor down-staging and resection with similar survival rates after surgery and a decrease in the rate of margin-positive resections (36–38). The resection rates of patients with BR-PDAC that ultimately undergo pancreatectomy after neoadjuvant therapy range from 47 to 76% with an associated 81 to 95% R0 resections (7, 16–20, 39). In this meta-analysis, despite the variability amongst the protocols, no difference was observed within the IPD cohort in the R0 rates among drug regimens with the majority of resected patients having negative margins.

The strength of the present study is that all the data were reappraised and reclassified as BR-PDAC at an IPD level according to the radiological criteria. However, the main limitation of the present study is the heterogeneity of the included studies, although only one study is a retrospective study. In particular, some studies failed to report eligibility criteria for neo-adjuvant treatment expect that for the BR-PDAC stage (18, 20). A second limitation is related to the evaluation of the safety and tolerability of neoadjuvant therapy. Characteristically, retrospective studies did not include information about dose reduction or discontinuation of study drugs due to adverse events. Finally, there was paucity of data on adjuvant or second line chemotherapy. Therefore, we were not able to assess their impact on the survival outcomes.

In conclusion, to the best of our knowledge, the present meta-analysis is the first evidence-based data aggregation of gemcitabine-based Neoadjuvant Treatment in BR-PDAC. The results support the use of GEM-NAT for BR-PDAC in routine practice to select patients where surgery may contribute the most benefit. Moreover, the general tolerability of GEM-NAT may improve the rate of patients undergoing surgery following NAT and receiving adjuvant treatment related to lower post-operative complications and/or decline in the functional status (40, 41).

## Data Availability Statement

The datasets presented in this article are not readily available due to maintaining patient confidentiality. Requests to access the datasets should be directed to giovinazzo_francesco@live.com.

## Author Contributions

FG and FS contributed to study design, data collection, data analyses, data interpretation, writing, and reviewing. HA and FS did the literature search and figures. J-YJ, EV, GT, CE, YH, SC, CK, MZ, SY, SH, JR, CT, YN, and MA contributed to data collection and reviewing of the report. All authors contributed to the article and approved the submitted version.

## Conflict of Interest

The authors declare that the research was conducted in the absence of any commercial or financial relationships that could be construed as a potential conflict of interest.
