# Supplementary figures and images for: Gemcitabine-Based Neoadjuvant Treatment in Borderline Resectable Pancreatic Ductal Adenocarcinoma: A Meta-Analysis of Individual Patient Data
Source: Front Oncol. 2020 Aug 11;10:1112. doi: 10.3389/fonc.2020.01112 (PMC7431761; doi:10.3389/fonc.2020.01112)

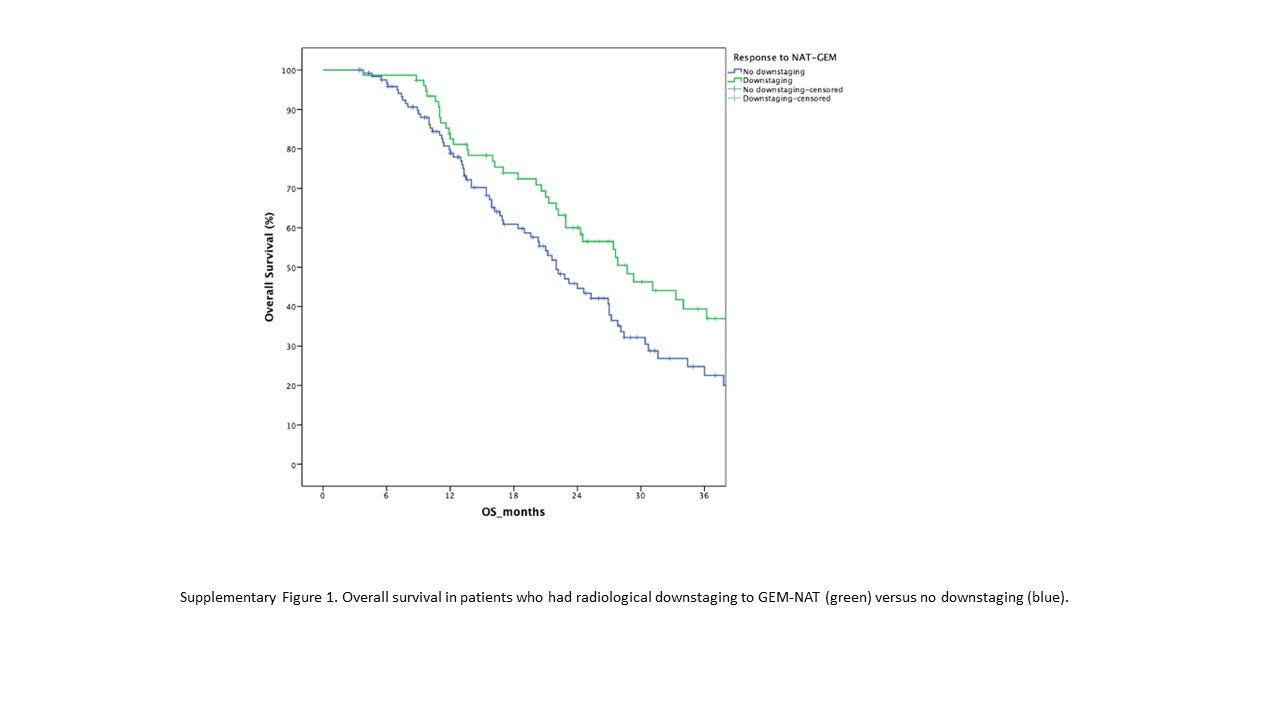

Supplement: Supplementary file 1 [file Image_1.JPEG]
